# Prospective Registry and Meta‐Analysis of Particle Therapy for Hepatocellular Carcinoma: Clinical Outcomes and Real‐World Impact

**DOI:** 10.1002/cam4.71639

**Published:** 2026-02-20

**Authors:** Masashi Mizumoto, Kei Shibuya, Kazuki Terashima, Hiromitsu Iwata, Takashi Saito, Shingo Toyama, Takashi Ogino, Masao Murakami, Tatsuya Ohno, Yoshitaka Sato, Tetsuo Akimoto, Hiroyuki Katoh, Masaru Wakatsuki, Takahiro Waki, Norio Katoh, Fuki Koizumi, Masayuki Araya, Tsuyoshi Onoe, Masaru Takagi, Tomoaki Okimoto, Hiroyuki Ogino, Haruko Numajiri, Yoshiyuki Shioyama, Takayuki Hashimoto, Hisateru Ohba, Shoji Kubo, Kiyoshi Hasegawa, Kazushi Maruo, Hidefumi Aoyama, Hideyuki Sakurai

**Affiliations:** ^1^ Department of Radiation Oncology University of Tsukuba Ibaraki Japan; ^2^ Department of Radiation Oncology Gunma University Graduate School of Medicine Maebashi Japan; ^3^ Department of Radiology Hyogo Ion Beam Medical Center Tatsuno Hyogo Japan; ^4^ Department of Radiation Oncology Nagoya City University West Medical Center Nagoya Aichi Japan; ^5^ Ion Beam Therapy Center SAGA HIMAT Foundation Saga Japan; ^6^ Medipolis Proton Therapy and Research Center Kagoshima Japan; ^7^ Department of Radiation Oncology Southern Tohoku Proton Therapy Center Fukushima Japan; ^8^ Proton Therapy Center Fukui Prefectural Hospital Fukui Japan; ^9^ Department of Radiation Oncology National Cancer Center Hospital East Chiba Japan; ^10^ Department of Radiation Oncology Kanagawa Cancer Center Yokohama Kanagawa Japan; ^11^ QST Hospital, National Institute for Quantum Science and Technology Chiba Japan; ^12^ Department of Radiology Tsuyama Chuo Hospital Okayama Japan; ^13^ Department of Radiation Oncology Hokkaido University Faculty of Medicine Sapporo Hokkaido Japan; ^14^ Proton Therapy Center, Aizawa Hospital Nagano Japan; ^15^ Division of Radiation Oncology Radiation and Proton Therapy Center, Shizuoka Cancer Center Shizuoka Japan; ^16^ Department of Radiation Oncology Sapporo Teishinkai Hospital Sapporo Japan; ^17^ Department of Hepato‐Biliary‐Pancreatic Surgery Osaka Metropolitan University Graduate School of Medicine Osaka Japan; ^18^ Hepato‐Biliary‐Pancreatic Surgery Division, Department of Surgery, Graduate School of Medicine The University of Tokyo Tokyo Japan; ^19^ Department of Biostatistics, Faculty of Medicine University of Tsukuba Ibaraki Japan

**Keywords:** HCC, hepatocellular carcinoma, meta‐analysis, particle therapy, prospective, systematic review

## Abstract

**Background and Aims:**

This study aimed to evaluate the clinical efficacy of particle therapy for hepatocellular carcinoma (HCC) by integrating a prospective registry and a systematic meta‐analysis. Our findings also reflect the real‐world impact of this evidence, which contributed to the national health insurance approval of particle therapy for HCC tumors ≥ 4 cm in Japan.

**Methods:**

Patients who received particle therapy for HCC from May 2016 to June 2018 were registered. Ninety studies (25 particle therapy, 26 non‐SBRT, 36 SBRT, 3 studies reporting multiple modalities) were selected.

**Results:**

A total of 836 cases (proton beam therapy 576, carbon therapy 260) were examined. The median overall survival (OS) was 53.7 months (95% CI 47.4‐NA). The 1‐, 2‐, 3‐ and 4‐year OS rates were 85.2% (95% CI 82.6%–87.4%), 71.4% (68.1%–74.4%), 60.5% (56.9%–63.9%), and 53.5% (49.1%–57.7%), respectively; and the 1‐, 2‐, 3‐ and 4‐year local recurrence rates were 3.5% (2.1%–4.9%), 8.8% (6.3%–10.8%), 12.0% (9.3%–14.8%), and 13.6% (10.5%–16.7%), respectively. In the meta‐analysis and registry data, the 1‐, 2‐, and 3‐year OS rates of particle therapy and SBRT for small HCC (< 4 cm) were 90.0%/87.7% (*p* = 0.4788), 75.3%/73.6% (*p* = 0.6724), and 62.8%/63.5% (*p* = 0.9771), respectively; and the 1‐, 2‐, and 3‐year OS rates of particle therapy, SBRT and non‐SBRT for large HCC (≥ 4 cm) were 81.1%/62.0% (*p* = 0.0032)/66.8% (*p* = 0.0021), 65.4%/38.1% (*p* = 0.0001)/38.4% (*p* = 0.0001), and 50.4%/31.8% (*p* = 0.0001)/25.9% (*p* = 0.0001), respectively.

**Conclusions:**

A prospective registry study and meta‐analysis indicated that particle therapy is a better treatment modality than SBRT for large HCC. Particle therapy and SBRT gave similar outcomes for small HCC. These findings contributed to the adoption of particle therapy for tumors ≥ 4 cm under Japan's national health insurance, highlighting its real‐world impact.

## 1 Introduction

Surgical resection, radiofrequency ablation (RFA) and liver transplantation are standard curative treatments for hepatocellular carcinoma (HCC) ([1], [2]) and are selected according to liver function and tumor size and number. Particle therapy has excellent dose concentration and is likely to be effective in reducing the risk of late adverse events and in enabling adequate dose escalation for tumors requiring high‐dose irradiation [3, 4]. These advantages are particularly relevant for hepatocellular carcinoma (HCC), where preservation of liver function and high‐dose irradiation are both critical. Our previous registry study indicated that proton beam therapy (PBT) for HCC could achieve a local control rate of about 90% [5]. In a systematic review of the overall survival (OS) rate of HCC after PBT and photon radiotherapy [6], Qi et al. found results that were equivalent to stereotactic body radiation therapy (SBRT) and better than three‐dimensional conformal radiation therapy (non‐SBRT). However, this analysis did not consider factors such as tumor size or the presence or absence of tumor vascular invasion, and thus, does not provide a clear answer as to which conditions are appropriate for use of particle therapy. To examine this issue, in this study we compared the results of prospective registry data for PBT and carbon therapy for HCC in Japan with those of particle therapy, SBRT and non‐SBRT (3D‐CRT/IMRT/VMAT) calculated from a meta‐analysis.

## 2 Registry Data

Patients who received PBT or carbon therapy from May 2016 to June 2018 were registered in a database. This analysis used the national multi‐institutional registry curated to support a health‐insurance listing dossier that was finalized in April 2022. For that process, participating institutions pre‐specified a common data‐lock and follow‐up cut‐off of 31 December 2018 to align follow‐up windows and the timeline for centralized auditing. Although additional cases (2019–2022) were accrued, site‐level source verification, covariate harmonization (e.g., staging and PVTT classification), and central audit were incomplete at the time of our analysis; therefore, we restricted the present analysis to patients treated between May 2016 and June 2018. We acknowledge that equal follow‐up is not required for time‐to‐event analyses; our choice reflects data completeness, governance, and cohort consistency rather than a statistical requirement.

The study was performed by the Hepatocellular Cancer Working Group in the Particle Beam Therapy Committee and Subcommittee of the Japanese Society for Radiation Oncology (JASTRO). The treatment results for PBT have already been reported [5]. A total of 836 cases were registered in the database, including 576 patients treated with PBT and 260 treated with carbon therapy. The characteristics of these patients are shown in Supplement 1; briefly, the median age was 73 years, most patients had preserved liver function (Child–Pugh A), and the median tumor diameter was 35 mm. At final follow‐up, 505 patients were alive and 331 had died. The median follow‐up period for survivors was 37 months (0–58 months). The median overall survival (OS) time of the 836 patients was 53.7 months (95% CI 47.4‐NA) and the 1‐, 2‐, 3‐ and 4‐year OS rates were 85.2% (95% CI 82.6%–87.4%), 71.4% (68.1%–74.4%), 60.5% (56.9%–63.9%), and 53.5% (49.1%–57.7%), respectively. Recurrence was observed in 544 cases, including local recurrence in 72 cases. The 1‐, 2‐, 3‐ and 4‐year local recurrence rates were 3.5% (2.1%–4.9%), 8.8% (6.3%–10.8%), 12.0% (9.3%–14.8%), and 13.6% (10.5%–16.7%), and the 1‐, 2‐, 3‐ and 4‐year death without local recurrence rates were 3.5% (2.2%–4.9%), 5.3% (3.6%–7.0%), 6.8% (4.7%–8.9%), and 8.0% (5.6%–10.5%), respectively. The OS, local recurrence and death without local recurrence rates are shown in Supplement 2a and 2b.

**FIGURE 1:**
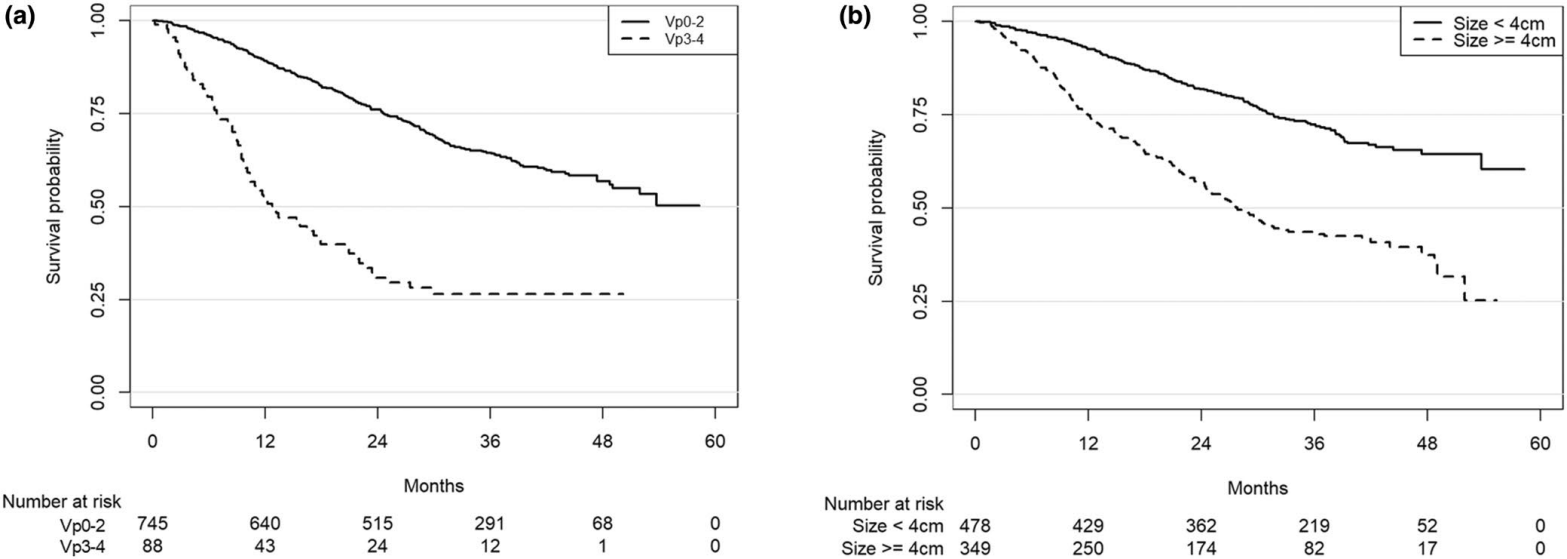
(a) Overall survival stratified by the extent of portal vein tumor thrombus (PVTT): Vp0–2 (no PVTT to second‐order branch involvement) versus Vp3–4 (first‐order branch/main trunk involvement). Numbers at risk are shown below. (b) Overall survival rates divided by tumor size (< 4 cm vs. 4 cm or more).

Survival rates were also compared by portal vein tumor thrombosis (PVTT) progression (Vp0–2 vs. Vp3–4). The median OS times (Vp0–2/Vp3–4) were NA/12.7 months (95% CI 9.7–20.6) and the 1‐, 2‐, 3‐ and 4‐year OS rates (Vp0–2/Vp3–4) were 89.1% (95% CI 86.6%–91.1%)/51.9% (40.8%–61.9%), 76.1% (72.8%–79.0%)/30.9% (21.3%–41.0%), 64.5% (60.7%–68.0%)/26.5% (17.3%–36.5%), and 56.8% (52.1%–61.3%)/26.5% (17.3%–36.5%), respectively. The OS rates in Vp0–2 and Vp3–4 cases are shown in Figure 1a.

Survival was also examined for cases with tumor sizes of < 4 vs. ≥ 4 cm. The median OS times (< 4/≥ 4 cm) were NA/27.8 months (95% CI 24.5–31.7) and the 1‐, 2‐, 3‐ and 4‐year OS rates were 92.6% (95% CI 89.8%–94.6%)/74.8% (69.8%–79.1%), 81.8% (78.0%–85.0%)/56.7% (51.2%–61.9%), 72.3% (67.8%–76.3%)/43.6% (37.9%–49.1%), and 64.4% (58.8%–69.5%)/37.4% (30.3%–44.5%), respectively. The OS rates in cases with tumor sizes of < 4 and ≥ 4 cm are shown in Figure 1b.

## 3 Meta‐Analysis

### 3.1 Selection Criteria

Comparator definition. In the meta‐analysis, the photon comparator was defined as non‐SBRT, encompassing 3D‐CRT, IMRT, and VMAT techniques delivered in ≥ 9–16 fractions (e.g., 15‐fraction ablative regimens). SBRT was defined as 3–6 fractions. Studies with mixed schedules (e.g., Sanford) were assigned according to the dominant fractionation pattern to avoid technique‐based misclassification; IMRT indicates *intensity‐modulated radiotherapy* and VMAT indicates *volumetric modulated arc therapy*.

The review was conducted in compliance with the Preferred Reporting Item for Systematic Reviews and Meta‐Analysis (PRISMA) guidelines and recommendations [7]. Only English language articles were included. All retrieved articles were screened by two reviewers. The inclusion criteria were: (1) clinically diagnosed primary HCC, (2) received radical radiotherapy (non‐SBRT, SBRT or particle therapy), radical radiotherapy was defined as treatment delivering ≥ 50 Gy (RBE) to the primary lesion including margins, with no active disease outside the irradiation field, and with curative intent for tumor control or survival improvement. Palliative cases were excluded, [3] curative treatment other than radiotherapy (surgery, RFA or liver transplantation) not used concurrently (curative treatment for recurrence was acceptable), [4] median survival or survival rate with radiotherapy can be confirmed in the manuscript, and [5] ≥ 10 treatment results specified” referred to studies including at least 10 patients with available data for key clinical outcomes (overall survival, local control, or progression‐free survival).

We selected manuscripts published between 2000 and 2016 that met the five eligibility criteria and were included in the liver cancer clinical practice guidelines. In addition to PubMed searches, we also reviewed studies referenced in the hepatocellular carcinoma clinical practice guidelines issued by the Japan Society of Hepatology (JSH) and related collaborative groups. This approach ensured that important particle therapy studies not retrievable through PubMed alone were captured, thereby providing a comprehensive and reliable literature selection. Manuscripts from 2016 to 2020 that met the same five criteria were additionally selected from 1303 articles identified in a PubMed search using “Radiotherapy” AND “Hepatocellular carcinoma”. A total of 130 manuscripts (38 particle therapy, 47 SBRT, 45 non‐SBRT) from 2000 to 2020 were initially identified using these methods. From these manuscripts, 90 (25 particle therapy, 36 SBRT, 26 non‐SBRT RT, 3 multiple modalities) [8, 9, 10, 11, 12, 13, 14, 15, 16, 17, 18, 19, 20, 21, 22, 23, 24, 25, 26, 27, 28, 29, 30, 31, 32, 33, 34, 35, 36, 37, 38, 39, 40, 41, 42, 43, 44, 45, 46, 47, 48, 49, 50, 51, 52, 53, 54, 55, 56, 57, 58, 59, 60, 61, 62, 63, 64, 65, 66, 67, 68, 69, 70, 71, 72, 73, 74, 75, 76, 77, 78, 79, 80, 81, 82, 83, 84, 85, 86, 87, 88, 89, 90, 91, 92, 93, 94, 95, 96, 97] were finally selected after excluding manuscripts with significant bias in patient background and overlapping publication periods from the same institute. The manuscript selection process is shown in Supplement 3. Data were obtained from each manuscript for authors, year of publication, country, study design, number of patients, number of deaths, follow‐up period, median survival time, 1‐year OS rate, 2‐year OS rate, 3‐year OS rate, tumor size, number of PVTT cases, and irradiation methods (particle therapy, SBRT, non‐SBRT). For example, in Sanford 2019, proton therapy was delivered with three‐dimensional passively scattered protons, while photon therapy was planned with IMRT or VMAT. If median survival time (MST) and the 1‐, 2‐ and 3‐year OS rates were not specified in the text, these data were estimated from figures.

### 3.2 Statistical Techniques

Random‐effects meta‐analyses of 1‐, 2‐, and 3‐year OS rates and MST were performed for each modality, and forest plots were drawn. For studies with missing accuracy data, missing values were imputed using information on the number of cases, risk set size at each year, and mean dropout rate. Heterogeneity in each meta‐analysis was evaluated by I^2^ statistics. Random‐effects meta‐regressions with modality as an explanatory variable were also used for each outcome to compare among the modalities. All analyses were performed using R software (R Core Team, Vienna, Austria) and its meta package [98].

## 4 Results

**FIGURE 2:**
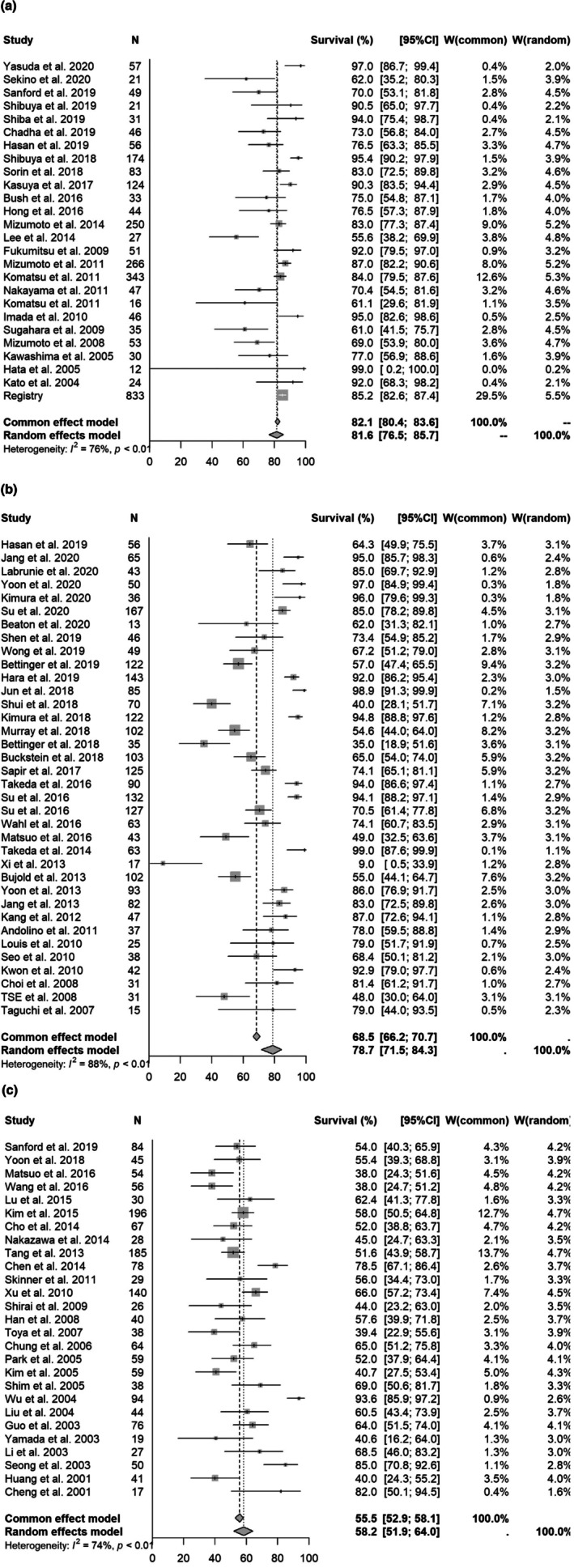
Forest plot for each modality of 1‐year overall survival rate (All selected manuscript). (a) 1‐year overall survival rate in all selected studies (particle therapy). (b) 1‐year overall survival rate of all selected manuscripts (SBRT). (c) 1‐year overall survival rate of all selected manuscripts (3DCRT).

Meta‐analysis was first performed using all selected manuscripts and registry data. The 1‐, 2‐, and 3‐year OS rates for particle therapy, SBRT and non‐SBRT were [1 year OS] 81.6% (95% CI 76.5%–85.7%)/78.7% (71.5%–84.3%, *p* = 0.2432)/58.2% (51.9%–64.0%, *p* = 0.0001); [2‐year OS] 65.9% (60.9%–70.4%)/64.8% (55.9%–72.3%, *p* = 0.6175)/32.3% (27.3%–37.3%, *p* = 0.0001); and [3‐year OS] 55.4% (51.1%–59.4%)/55.9% (47.6%–63.4%, *p* = 0.9272)/20.7% (15.7%–26.2%, *p* = 0.001), respectively. Forest plots for each modality are shown in Figure 2 and Supplement 4 and 5. In multivariate analysis, non‐SBRT and presence of PVTT were significantly associated with a poor prognosis (Table 1).

**TABLE 1:** Meta‐regressions of potential predictive factors for 1‐, 2‐ and 3‐year overall survival.

Factors	Coefficient	SE	Lower CL	Upper CL	Z value	*p* value
All studies						
1‐year OS						
SBRT	0.437	0.196	0.054	0.821	2.235	0.025
3D‐CRT	0.452	0.213	0.034	0.869	2.121	0.034
Tumor size	0.007	0.004	0.000	0.014	1.939	0.053
PVTT/IVCTT	0.014	0.002	0.009	0.018	6.292	< 0.001
2‐year OS						
SBRT	0.301	0.169	−0.030	0.632	1.782	0.075
3D‐CRT	0.605	0.178	0.256	0.953	3.402	0.001
Tumor size	0.007	0.003	0.001	0.014	2.209	0.027
PVTT/IVCTT	0.007	0.002	0.003	0.011	3.250	0.001
3‐year OS						
SBRT	0.152	0.152	−0.145	0.449	1.005	0.315
3D‐CRT	0.613	0.185	0.251	0.976	3.314	0.001
Tumor size	0.005	0.003	−0.001	0.011	1.587	0.113
PVTT/IVCTT	0.008	0.002	0.004	0.012	3.727	< 0.001
Excluding studies with only PVTT/IVCTT						
1‐year OS						
SBRT	0.266	0.249	−0.222	0.755	1.069	0.285
3D‐CRT	0.421	0.330	−0.227	1.068	1.273	0.203
Tumor size	0.015	0.005	0.004	0.025	2.663	0.008
2‐year OS						
SBRT	0.260	0.185	−0.104	0.623	1.401	0.161
3D‐CRT	0.596	0.240	0.126	1.065	2.484	0.013
Tumor size	0.011	0.004	0.003	0.019	2.576	0.010
3‐year OS						
SBRT	0.084	0.160	−0.230	0.398	0.523	0.601
3D‐CRT	0.487	0.223	0.049	0.925	2.180	0.029
Tumor size	0.009	0.004	0.002	0.017	2.492	0.013

Abbreviations: CL, confidence limit; IVCTT, inferior vena cava tumor thrombosis; MST, median survival time; OS, overall survival; PVTT, portal vein tumor thrombosis; SBRT, stereotactic body radiation therapy; 3D‐CRT, three‐dimensional conformal radiation therapy; SE, standard error.

**FIGURE 3:**
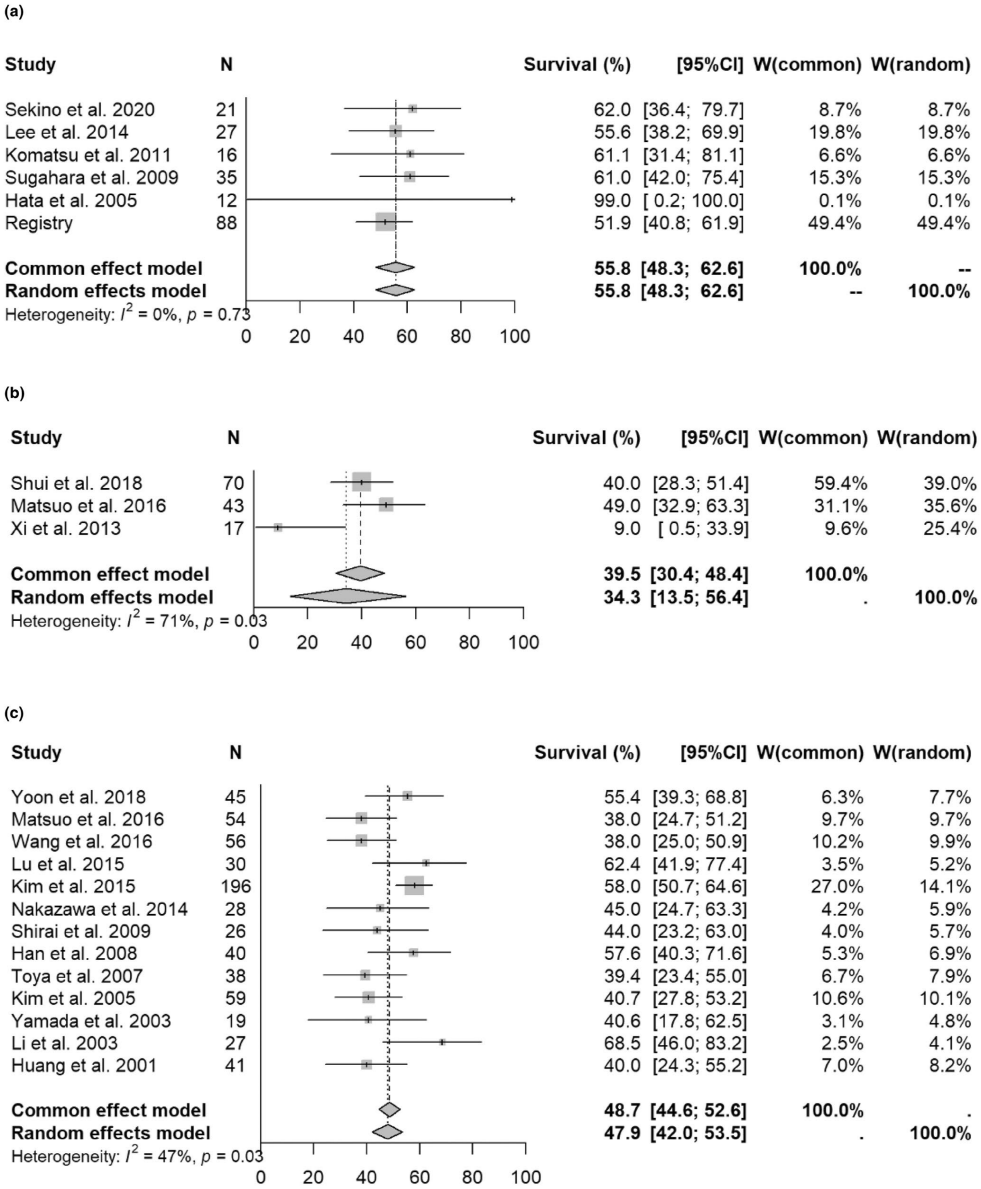
Forest plot for each modality of 1‐year overall survival rate (PVTT/IVCTT). (a) 1‐year overall survival rate for studies with PVTT/IVCTT (particle therapy). (b) 1‐year overall survival rate focused on PVTT/IVCTT (SBRT). (c) 1‐year overall survival rate focused on PVTT/IVCTT (3DCRT).

In studies with PVTT/IVCTT(inferior vena cava tumor thrombosis), the 1‐ and 2‐year OS rates for particle therapy, SBRT and non‐SBRT were [1 year OS] 55.8% (48.3%–62.6%)/34.3% (13.5%–56.4%, *p* = 0.0166)/47.9% (42.0%–53.5%, *p* = 0.1092); and [2‐year OS] 45.3% (37.4%–52.8%)/no data (no more than two studies)/23.1% (19.6%–26.9%, *p* = 0.0001), respectively. Forest plots for each modality are shown in Figure 3 and Supplement 6. The results showed a trend toward better outcomes with particle therapy, but this was based only on a small amount of literature and registry data.

**FIGURE 4:**
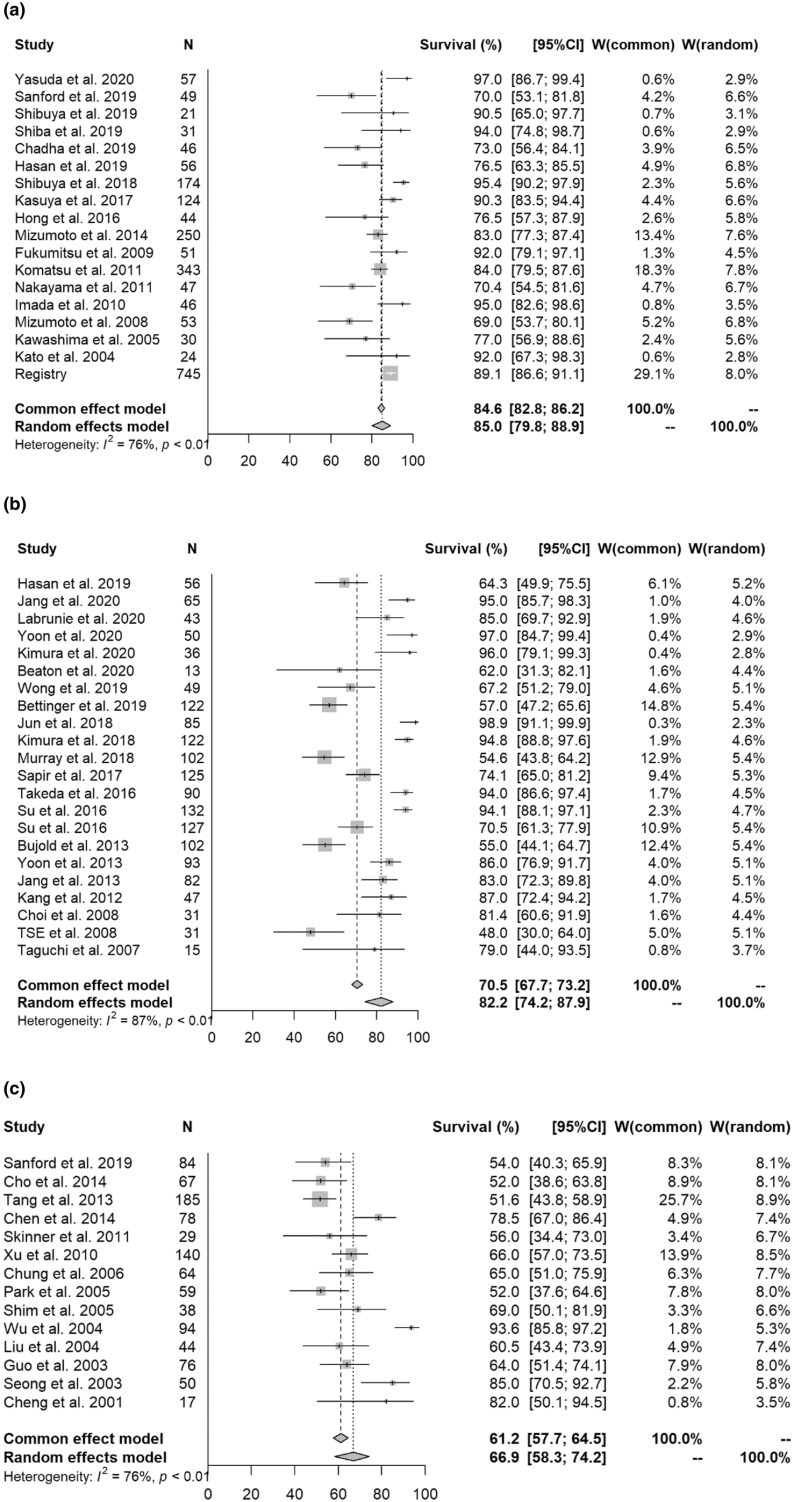
Forest plot for each modality of 1‐year overall survival rate (without PVTT/IVCTT). (a) 1‐year overall survival rate excluding studies with only PVTT/IVCTT (particle therapy). (b) 1‐year overall survival rate excluding only PVTT/IVCTT (SBRT). (c) 1‐year overall survival rate excluding only PVTT/IVCTT (3DCRT).

In an analysis excluding manuscripts with only PVTT/IVCTT. the 1‐, 2‐, and 3‐year OS rates for particle therapy, SBRT and non‐SBRT were [1 year OS] 85.0% (95% CI 79.8%–88.9%)/82.2% (74.2%–87.9%, *p* = 0.2858)/66.9% (58.3%–74.2%, *p* = 0.0013); [2‐year OS] 69.7% (65.2%–73.7%)/64.7% (51.2%–75.3%, *p* = 0.2081)/38.5% (32.4%–44.5%, *p* = 0.0001); and [3‐year OS] 56.8% (51.6%–61.7%)/56.7% (44.8%–66.9%, *p* = 0.7450)/26.2% (22.2%–30.4%, *p* = 0.001), respectively. Forest plots for each modality are shown in Figure 4 and Supplement 7 and 8. In multivariate analysis, non‐SBRT and large tumor size were significantly associated with a poor prognosis (Table 1).

**FIGURE 5:**
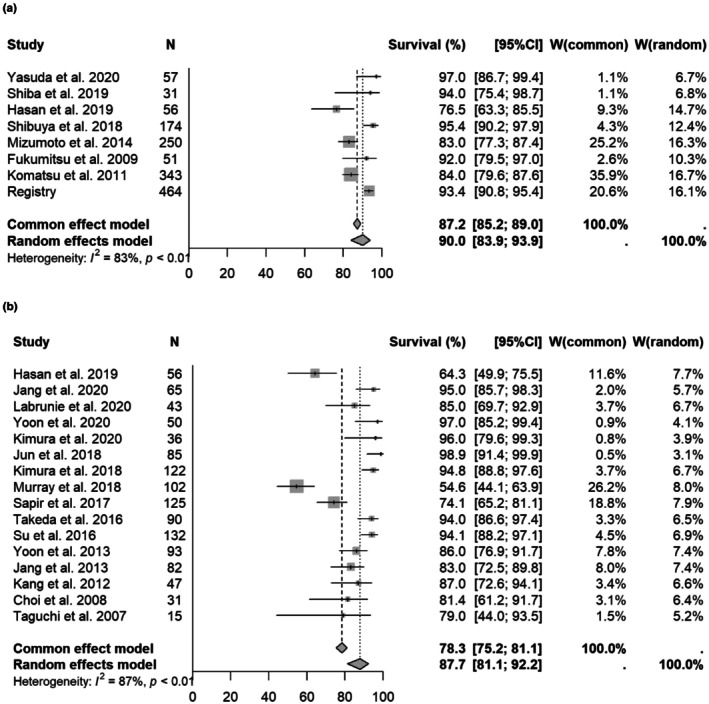
Forest plot for each modality of 1‐year overall survival rate (Small HCC). (a) 1‐year overall survival rate for small tumors (particle therapy). (b) 1‐year overall survival rate focused on small tumor (SBRT).

**FIGURE 6:**
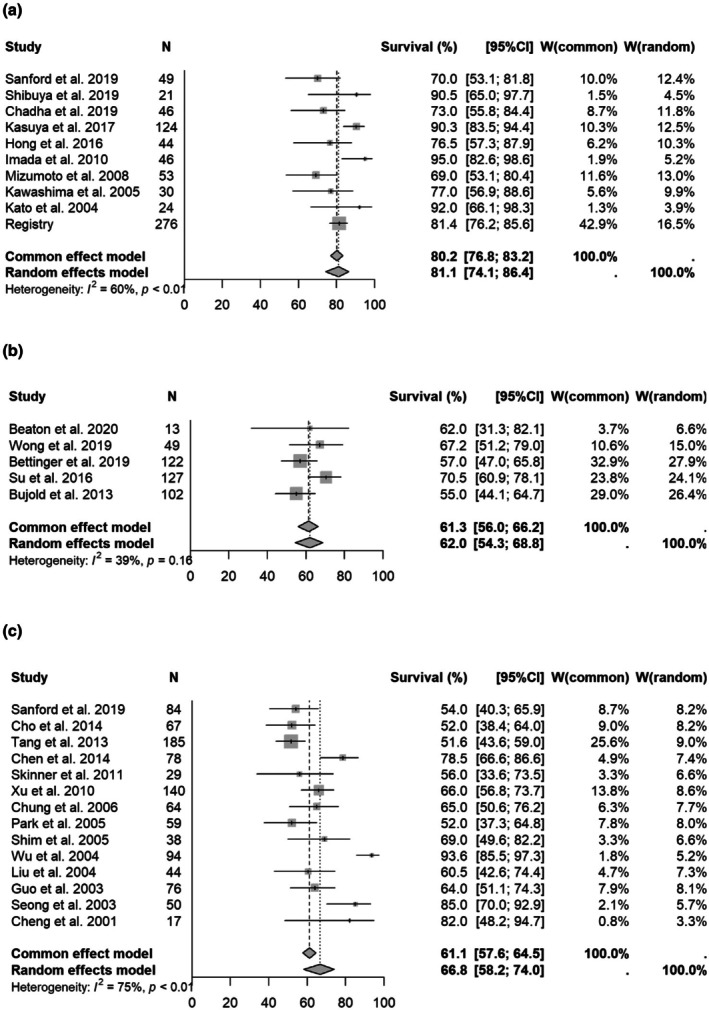
Forest plot for each modality of 1‐year overall survival rate (Large HCC). (a) 1‐year overall survival rate for large tumors (particle therapy). (b) 1‐year overall survival rate focused on large tumor (SBRT). (c) 1‐year overall survival rate focused on large tumor (3DCRT).

An analysis was also performed for studies with a median tumor size of < 4 and ≥ 4 cm, with articles describing PVTT/IVCTT‐only outcomes also excluded. In this analysis, the 1‐, 2‐, and 3‐year OS rates for particle therapy and SBRT for small HCC (< 4 cm) were [1 year OS] 90.0% (95% CI 83.9%–93.9%)/87.7% (81.1%–92.2%, *p* = 0.4788); [2 year OS] 75.3% (69.1%–80.4%)/73.6% (62.0%–82.2%, *p* = 0.6724); and [3 year OS] 62.8% (53.8%–70.5%)/63.5% (51.3%–73.3%, *p* = 0.9771), respectively. Forest plots for each modality are shown in Figure 5 and Supplement 9 and 10. Similarly, the 1‐, 2‐, and 3‐year OS rates for particle therapy, SBRT and non‐SBRT for large HCC (≥ 4 cm) were [1 year OS] 81.1% (95% CI 74.1%–86.4%)/62.0% (54.3%–68.8%, *p* = 0.0032)/66.8% (58.2%–74.0%, *p* = 0.0021); [2‐year OS] 65.4% (60.3%–70.0%)/38.1% (30.9%–45.2%, *p* = 0.0001)/38.4% (32.3%–44.4%, *p* = 0.0001); and [3‐year OS] 50.4% (46.2%–54.4%)/31.8% (20.1%–44.2%, p = 0.0001)/25.9% (21.9%–30.2%, p = 0.0001), respectively. Forest plots for each modality are shown in Figure 6 and Supplement 11 and 12.

## 5 Discussion

In this study, outcomes in the Japanese registry data were almost the same as those obtained in the meta‐analysis. Particle radiotherapy during the registry data period was performed according to the model policy proposed by JASTRO [99]. This policy only stipulates total dose and dose fractionation; dose constraints for risk organs and setting of margins for tumors are not stipulated. Thus, our results suggest that good outcomes can be obtained by performing irradiation prescribed by the model policy while retaining the tolerance dose for normal tissue.

Previous systematic reviews have found that OS rates are almost the same for PBT and SBRT, and lower for non‐SBRT [6, 100]. This trend was similar in the current meta‐analysis (Figure 2, Supplement 4, 5). However, in most reports, SBRT is used for tumors of 3 to 4 cm or less, particle therapy is used for those of 3 to 4 cm, and non‐SBRT is mainly used for tumors of ≥ 5 cm [8, 9, 10, 11, 12, 13, 14, 15, 16, 17, 18, 19, 20, 21, 22, 23, 24, 25, 26, 27, 28, 29, 30, 31, 32, 33, 34, 35, 36, 37, 38, 39, 40, 41, 42, 43, 44, 45, 46, 47, 48, 49, 50, 51, 52, 53, 54, 55, 56, 57, 58, 59, 60, 61, 62, 63, 64, 65, 66, 67, 68, 69, 70, 71, 72, 73, 74, 75, 76, 77, 78, 79, 80, 81, 82, 83, 84, 85, 86, 87, 88, 89, 90, 91, 92, 93, 94, 95, 96, 97]. An analysis of tumors < 4 cm showed no significant difference in survival between SBRT and particle therapy. This result suggests that both of these modalities can be expected to achieve high local control for small HCC, and that a similar OS rate is likely. Since there are few papers on non‐SBRT for tumors of median size ≤ 5 cm, comparison by meta‐analysis is difficult. However, since non‐SBRT generally delivers a lower irradiation dose to the tumor, it is likely that the outcomes will be poorer than those of particle therapy and SBRT.

The meta‐analysis of large HCC of median size ≥ 4 cm showed significantly better OS rates for particle therapy than for SBRT and non‐SBRT. Particle therapy is suitable for administering a high dose to the tumor while minimizing the dose to the normal liver, even if the tumor is large [100, 101]. Based on these characteristics, particle therapy may contribute to improving OS rates by preserving liver function while administering a high dose to the tumor. In our analysis of large tumors, SBRT and non‐SBRT gave similar treatment results, and non‐SBRT was mainly performed in combination with TACE. Our analysis was not designed to directly compare SBRT alone with TACE. Many of the SBRT studies included patients who also received TACE, reflecting common clinical practice. Therefore, while comparable outcomes were observed in large tumors (≥ 4 cm), these results should be interpreted with caution, as they may partly reflect the contribution of combined SBRT–TACE therapy rather than SBRT alone.

A meta‐analysis of PVTT/IVCTT also showed the superiority of particle therapy. In PVTT/IVCTT, the irradiation range tends to be wide because the extent of tumor development within the blood vessel is unclear and it is necessary to cover the area in which the tumor may have developed along the blood vessel. In addition, since PVTT/IVCTT is basically located in the central part of the liver surrounded by normal liver tissue, particle therapy is effective in preventing the spread of a low dose to the normal liver (9, 21, 26, 28, 31, 44, 58, 70–72, 74, 75, 79, 81, 82, 85, 90, 93, 97). However, there are only a few studies of particle therapy and SBRT for PVTT/IVCTT, and these have different patient backgrounds. Thus, a meta‐analysis considering patient background is needed as more studies become available.

We note that particle therapy was not compared with surgery or RFA, which are the current standard local treatments for HCC. Some recent reports have indicated that particle therapy is as effective as these treatments [17, 102, 103, 104]. Thus, Kim et al. concluded that PBT was not inferior to RFA for small HCC in a phase III trial [102]; Sekino et al. found similar treatment efficacy and adverse events with PBT and RFA in a propensity score matched analysis [103]; and Bush et al. found in a randomized clinical trial that PBT and TACE gave similar OS, but that PFS and LC were improved with PBT and administration of PBT required fewer hospitalizations [17, 104]. More comparative analyses of these treatments may contribute to further expansion of the indication of particle therapy for HCC.

In addition to the absence of a comparison of particle therapy with surgery and RFA, there are several further limitations in the study. Furthermore, the registry extract analyzed here was curated to support a national health‐insurance listing dossier that was finalized in April 2022, for which participating institutions pre‐specified a common data‐lock and follow‐up cut‐off of 31 December 2018 to align follow‐up windows and centralized auditing. Extending the window beyond 2018 would have required re‐initiating multi‐center data abstraction, renewed approvals, and fresh cross‐site audit—procedures beyond the scope of this study. Importantly, the cohort of patients treated between May 2016 and June 2018 is largely contemporaneous with, and in several instances more recent than, the comparator cohorts included in our meta‐analysis; therefore, data currency is unlikely to compromise interpretability. We agree that analyzing 2019–2022 cases would be valuable, and we plan a dedicated update once those entries complete site verification and central audit.

The first is that risk factors other than tumor size and PVTT/IVCTT were not considered. Although studies focusing exclusively on PVTT/IVCTT were excluded, a small proportion of patients with PVTT may still have been included in the analyzed cohorts. This factor was considered in our multivariate analysis, but residual confounding cannot be ruled out. The next point is that the tumor size subgroup analysis was based on study‐level median maximum tumor diameter, not on individual patient‐level data, which may limit the precision of the subgroup findings. In addition, only OS was evaluated. Since HCC often has repeated recurrence in the liver, multiple treatments are often performed before radiotherapy. Survival may depend on the timing of irradiation, and there is a need for evaluation using other indices, such as local control. Another limitation is that the registry data were limited to the period from 2016 to 2018, which corresponded to the initial phase of nationwide proton therapy registration in Japan immediately after insurance approval. The registry itself was launched in 2016, and for the present analysis, the registry data were limited to 2016–2018 because the data‐lock and follow‐up cut‐off was set at 31 December 2018 to align cross‐site verification and centralized auditing for the national insurance dossier finalized in April 2022, rather than as a statistical requirement for equal follow‐up. These data were also used as part of the evidence package for the expansion of national insurance coverage in 2022. Furthermore, although we confirmed that the Su 2016 and 2020 studies analyzed different patient cohorts (≤ 5 cm vs. > 5 cm tumors in 2016, and inoperable BCLC‐A stage in 2020), one of the participating institutions was shared across these studies. Therefore, a partial overlap of cases cannot be completely excluded, which may have influenced the pooled results. Within all of these limitations, our meta‐analysis showed no significant difference in OS with particle therapy and SBRT for small HCC tumors of < 4 cm, but particle therapy was shown to be more useful for large HCC tumors of ≥ 4 cm and PVTT/IVCTT studies. In this context, SBRT was selected as the comparator because it is an established, guideline‐recommended local therapy for unresectable HCC and is widely used in clinical practice. Comparisons with other local therapies such as RFA or TACE are also clinically important and will be the focus of future investigations. In addition, other treatment options such as Y‐90 radioembolization and systemic immunotherapies represent important modalities for unresectable HCC, and future work should evaluate particle therapy within this broader therapeutic context.

The registry data matched the results of the meta‐analysis under almost all conditions, suggesting that a highly accurate meta‐analysis was conducted.

## Author Contributions

Conception/design: Masashi Mizumoto, Kei Shibuyab, Hideyuki Sakuraia, provision of study material or patients: Kazuki Terashimac, Hiromitsu Iwatad, Takashi Saitoa, Shingo Toyamae, Takashi Oginof, Masao Murakamig, Tatsuya Ohnob, Yoshitaka Satoh, Tetsuo Akimotoi, Hiroyuki Katohj, Masaru Wakatsukik, Takahiro Wakil, Norio Katohm, Masayuki Arayan, Tsuyoshi Onoeo, Masaru Takagip, Tomoaki Okimotoc, Hiroyuki Oginod, Haruko Numajiria, Yoshiyuki Shioyamae Collection and/or assembly of data: Takayuki Hashimotom, Hiroyuki Oginod. Data analysis and interpretation: Masashi Mizumoto, Kazushi Maruos, Kei Shibuyab, Manuscript writing: Masashi Mizumoto, Kei Shibuyab, final approval of manuscript: Hidefumi Aoyamam, Shoji Kuboq, Kiyoshi Hasegawar, Hideyuki Sakuraia. All authors (1) made substantial contributions to the study concept or the data analysis or interpretation; (2) drafted the manuscript or revised it critically for important intellectual content; (3) approved the final version of the manuscript to be published; and (4) agreed to be accountable for all aspects of the work.

## Funding

This work was supported by JSPS KAKENHI Grant Number JP22K07685.

## Disclosure

The authors have nothing to report.

## Ethics Statement

This study protocol was reviewed and approved by the Ethical Review Board for Life Science and Medical Research, Hokkaido University Hospital (approval number 016–0106).

## Consent

Written informed consent was obtained from all participants in the study.

## Conflicts of Interest

The authors declare no conflicts of interest.

## Supporting information


**Data S1:** Characteristics of patients and tumors.

**Data S2a:** Overall survival rates for all patients.

**Data S2b:** Local recurrence rate for all patients.

**Data S3:** Manuscript selection process.

**Data S4:** Forest plot of 2‐year overall survival rate for each modality (all selected studies).

**Data S5:** Forest plot of 3‐year overall survival rate for each modality (all selected studies).

**Data S6:** Forest plot of 2‐year overall survival rate for each modality (PVTT/IVCTT).

**Data S7:** Forest plot of 2‐year overall survival rate for each modality (without PVTT/IVCTT).

**Data S8:** Forest plot of 3‐year overall survival rate for each modality (without PVTT/IVCTT).

**Data S9:** Forest plot of 2‐year overall survival rate for each modality (small HCC).

**Data S10:** Forest plot of 3‐year overall survival rate for each modality (small HCC).

**Data S11:** Forest plot of 2‐year overall survival rate for each modality (large HCC).

**Data S12:** Forest plot of 3‐year overall survival rate for each modality (large HCC).

## Data Availability

The data that support the findings of this study are available from the corresponding author upon reasonable request.
