# Supplementary material for: Prospective Registry and Meta‐Analysis of Particle Therapy for Hepatocellular Carcinoma: Clinical Outcomes and Real‐World Impact
Source: Cancer Med. 2026 Feb 20;15(3):e71639. doi: 10.1002/cam4.71639 (PMC12921530; doi:10.1002/cam4.71639)
Supplement: Supplementary file 1 — Data S1: Characteristics of patients and tumors. [file CAM4-15-e71639-s004.docx]

Supplement 1. Characteristics of patients and tumors.

| Characteristics | Number | % |
| --- | --- | --- |
| Age (years) | 21-98 | 73 (median) |
| Gender |  |  |
| Male | 636 | 76.1 |
| Female | 200 | 23.9 |
| Surgical indication |  |  |
| Operable | 223 | 26.7 |
| Inoperable | 613 | 73.3 |
| ECOG performance status |  |  |
| 0 | 676 | 80.9 |
| 1 | 126 | 15.1 |
| 2 | 24 | 2.9 |
| 3 | 10 | 1.1 |
| History of hepatitis |  |  |
| Yes | 682 | 81.6 |
| No | 154 | 18.4 |
| Child-Pugh class |  |  |
| A | 678 | 81.1 |
| B | 147 | 17.6 |
| C | 7 | 0.8 |
| Treatment methods |  |  |
| Proton beam therapy | 576 | 68.9 |
| Carbon therapy | 260 | 31.1 |
| Tumor size (mm) |  |  |
|  | 5-200 | 35 (median) |
| < 30 | 323 | 38.6 |
| 30-49 | 254 | 30.4 |
| 50-99 | 181 | 21.7 |
| ≥ 100 | 72 | 8.6 |
| Portal vein tumor thrombus |  |  |
| Vp 0-2 | 748 | 89.5 |
| Vp 3-4 | 88 | 10.5 |
| Prior treatment |  |  |
| Yes | 509 | 60.9 |
| No | 327 | 39.1 |
| Prior radiotherapy |  |  |
| Yes | 83 | 9.9 |
| No | 753 | 90.1 |
| Clinical stage |  |  |
| I | 375 | 44.9 |
| II | 250 | 29.9 |
| III | 196 | 23.4 |
| IV | 16 | 1.9 |

ECOG, Eastern Cooperative Oncology Group
