# Supplementary figures and images for: Prospective Registry and Meta‐Analysis of Particle Therapy for Hepatocellular Carcinoma: Clinical Outcomes and Real‐World Impact
Source: Cancer Med. 2026 Feb 20;15(3):e71639. doi: 10.1002/cam4.71639 (PMC12921530; doi:10.1002/cam4.71639)

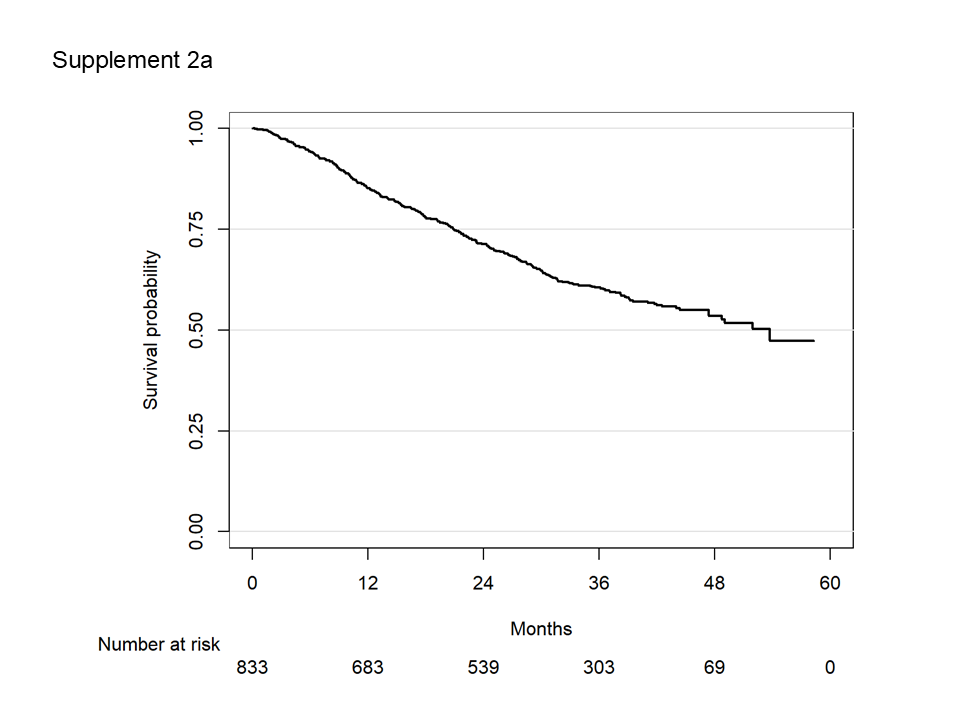

Supplement: Supplementary file 2 — Data S2a: Overall survival rates for all patients. [file CAM4-15-e71639-s008.tif]

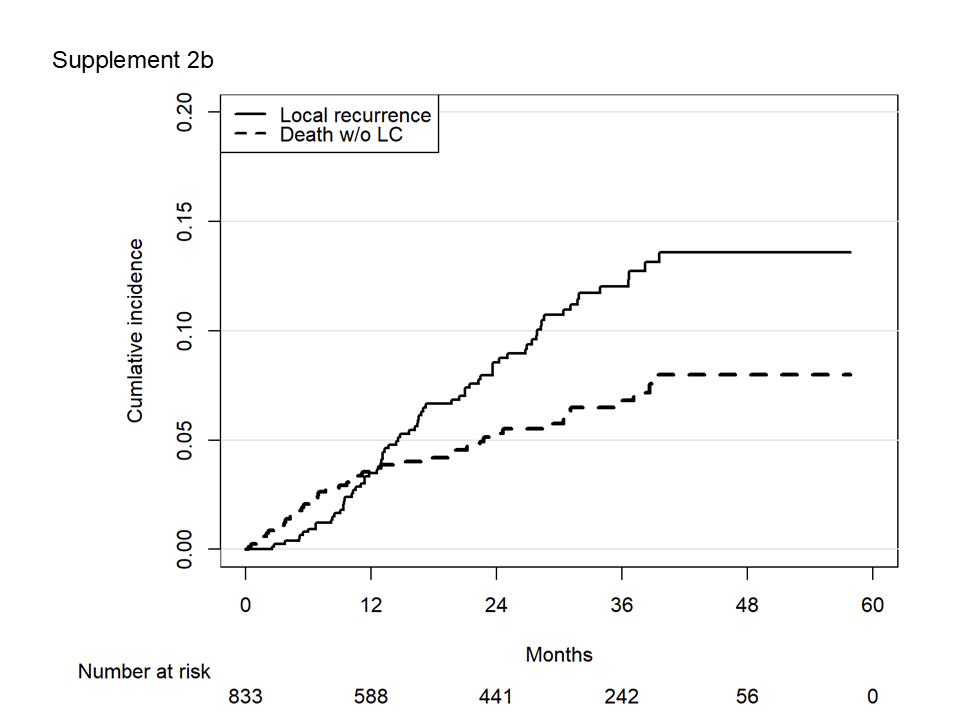

Supplement: Supplementary file 3 — Data S2b: Local recurrence rate for all patients. [file CAM4-15-e71639-s009.tif]

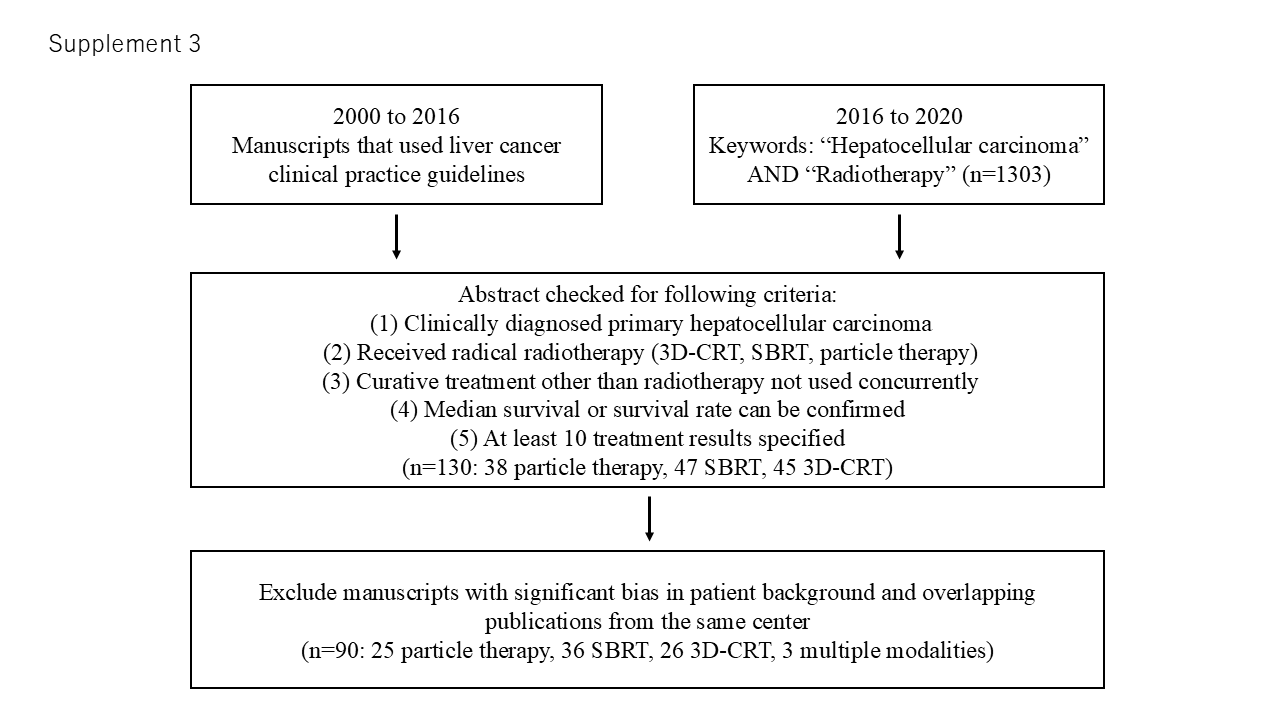

Supplement: Supplementary file 4 — Data S3: Manuscript selection process. [file CAM4-15-e71639-s005.tif]
