# Supplementary material for: Prospective Registry and Meta‐Analysis of Particle Therapy for Hepatocellular Carcinoma: Clinical Outcomes and Real‐World Impact
Source: Cancer Med. 2026 Feb 20;15(3):e71639. doi: 10.1002/cam4.71639 (PMC12921530; doi:10.1002/cam4.71639)

Supplement 5a. 3-year overall survival rate of all selected studies (particle therapy).


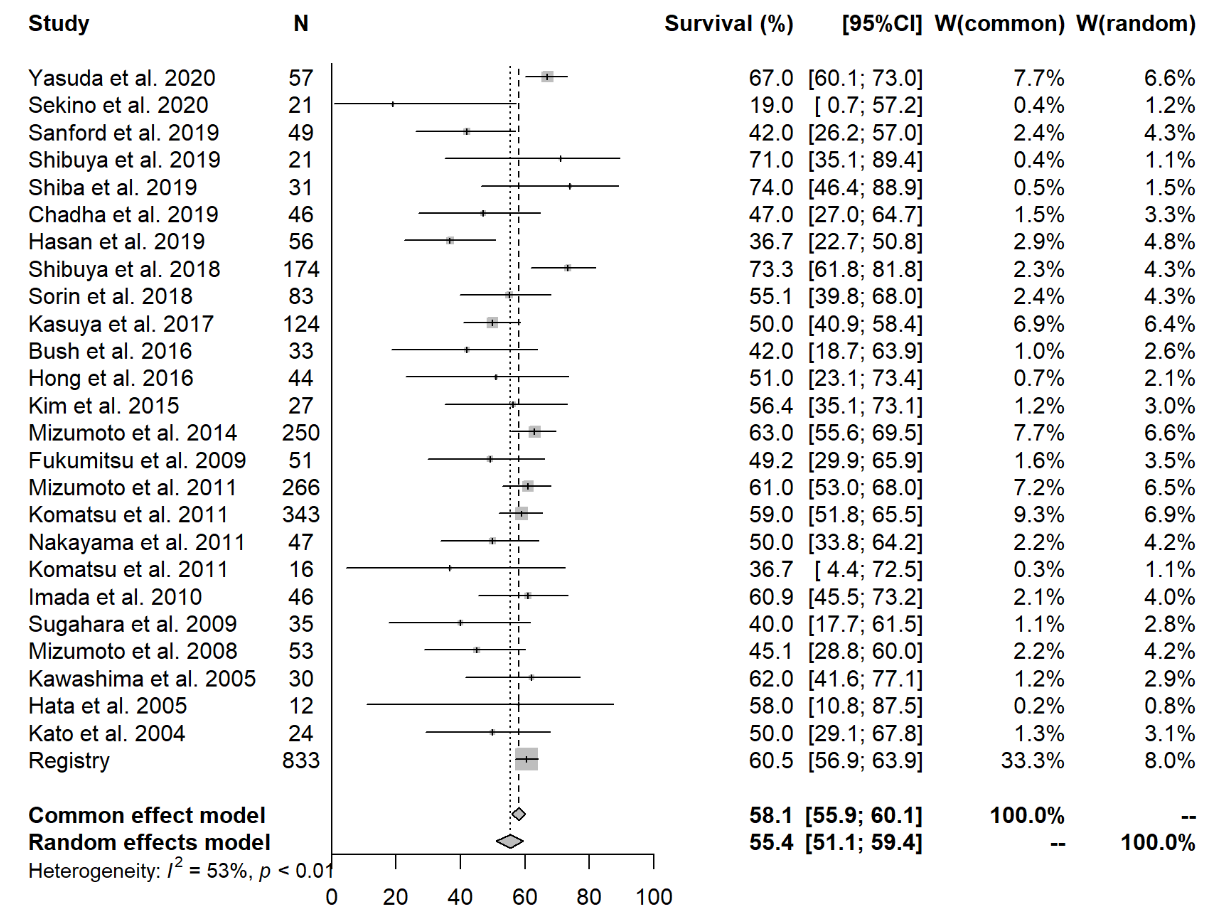


Supplement 5b. 3-year overall survival rate of all selected studies (SBRT).


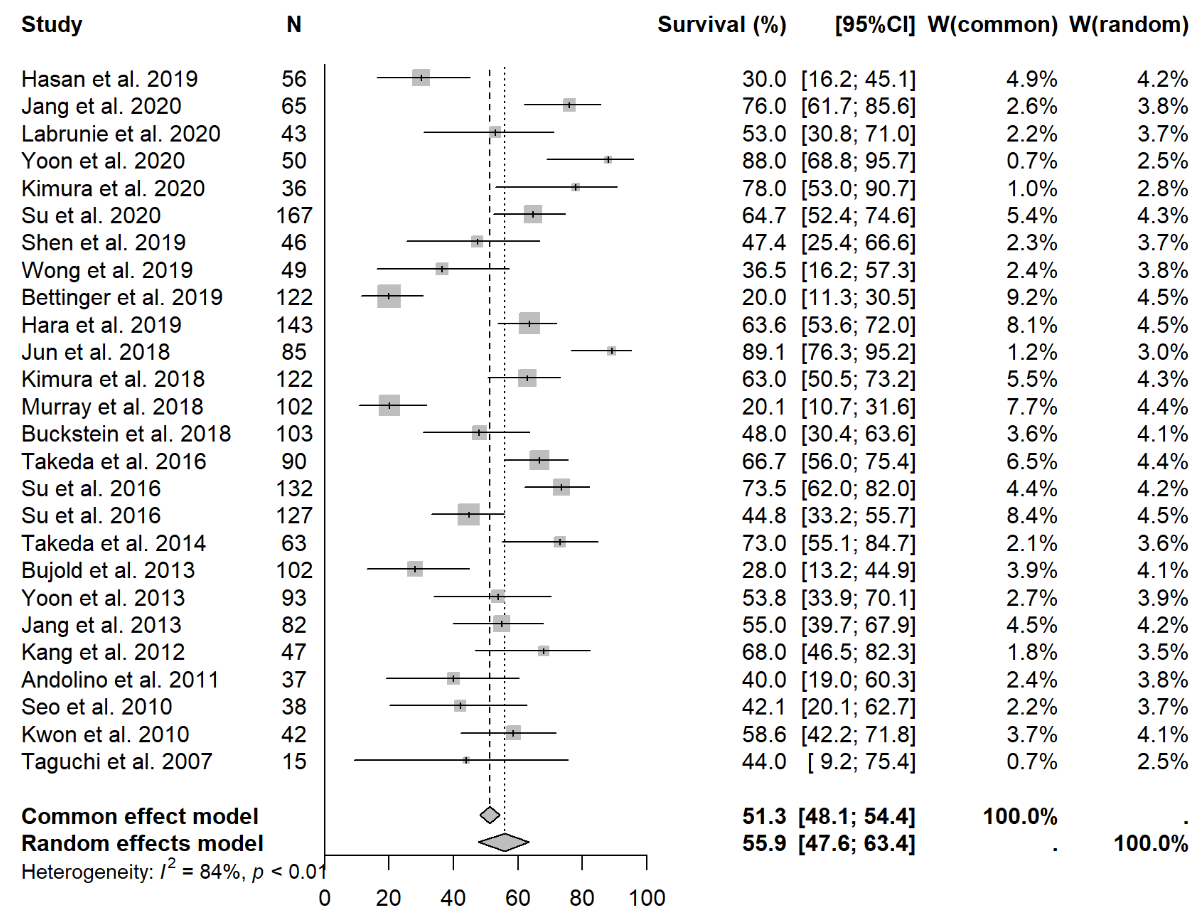


Supplement 5c. 3-year overall survival rate of all selected studies (3DCRT).


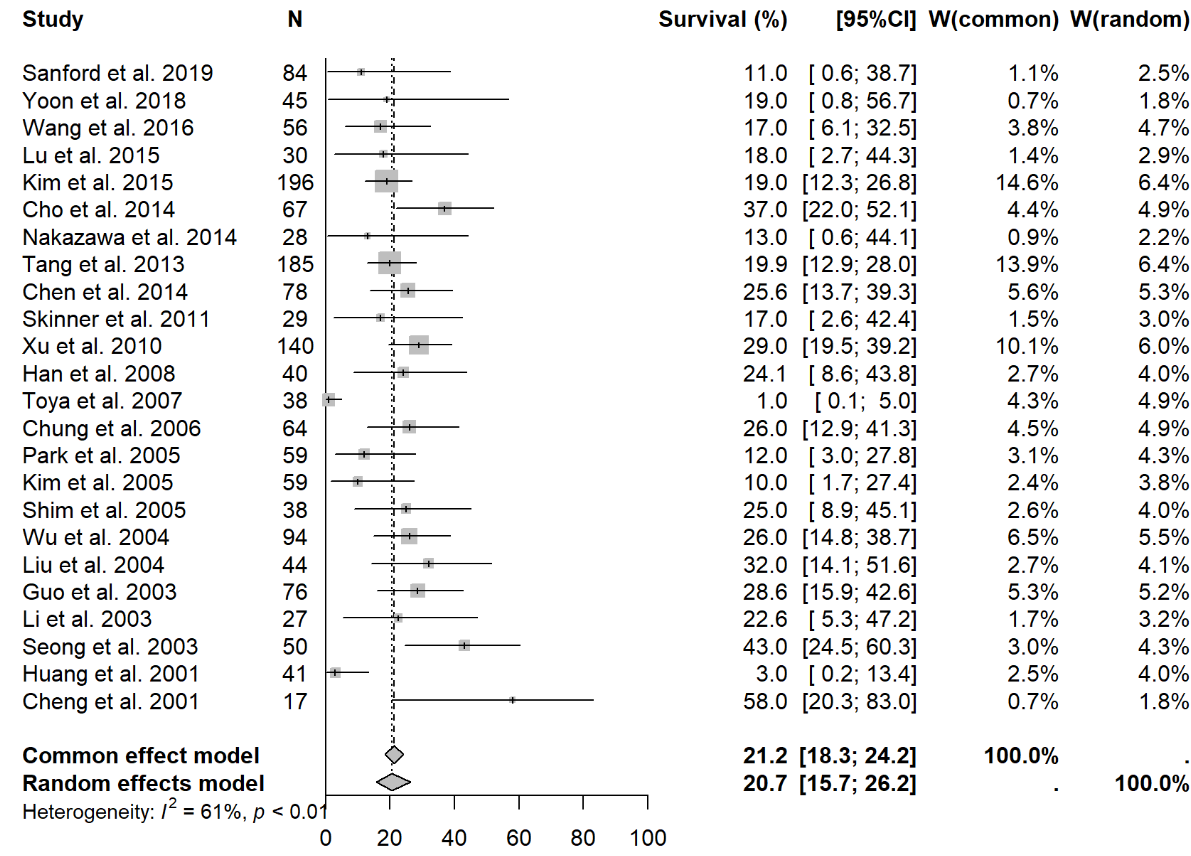

Supplement: Supplementary file 6 — Data S5: Forest plot of 3‐year overall survival rate for each modality (all selected studies). [file CAM4-15-e71639-s013.docx]
