# Supplementary material for: Prospective Registry and Meta‐Analysis of Particle Therapy for Hepatocellular Carcinoma: Clinical Outcomes and Real‐World Impact
Source: Cancer Med. 2026 Feb 20;15(3):e71639. doi: 10.1002/cam4.71639 (PMC12921530; doi:10.1002/cam4.71639)

Supplement 6a. 2-year overall survival rate for studies with PVTT/IVCTT (particle therapy).


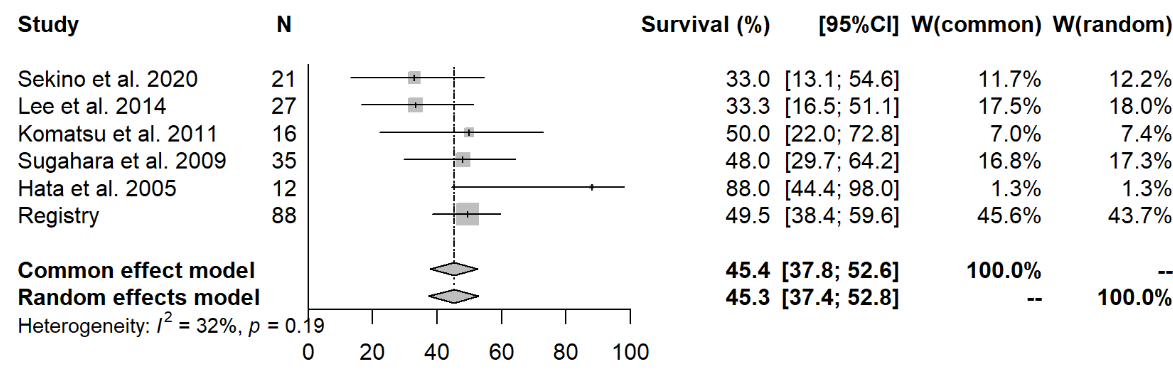


Supplement 6b. 2-year overall survival rate for studies with PVTT/IVCTT (3DCRT).


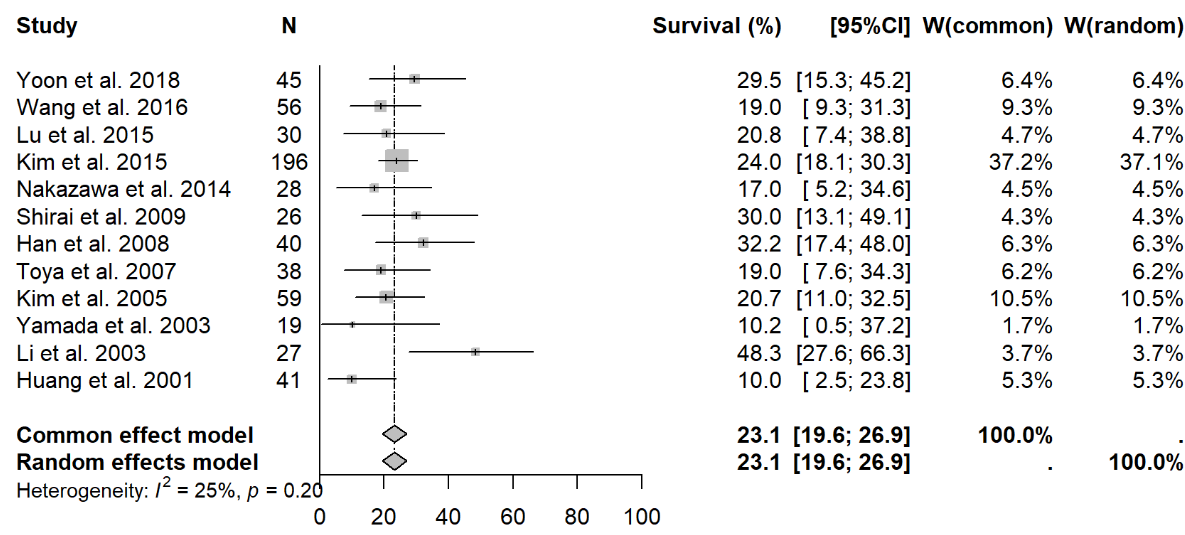

Supplement: Supplementary file 7 — Data S6: Forest plot of 2‐year overall survival rate for each modality (PVTT/IVCTT). [file CAM4-15-e71639-s006.docx]
