# Supplementary material for: Prospective Registry and Meta‐Analysis of Particle Therapy for Hepatocellular Carcinoma: Clinical Outcomes and Real‐World Impact
Source: Cancer Med. 2026 Feb 20;15(3):e71639. doi: 10.1002/cam4.71639 (PMC12921530; doi:10.1002/cam4.71639)

Supplement 11a, 2-year overall survival rate focused on large tumor (Particle Therapy).


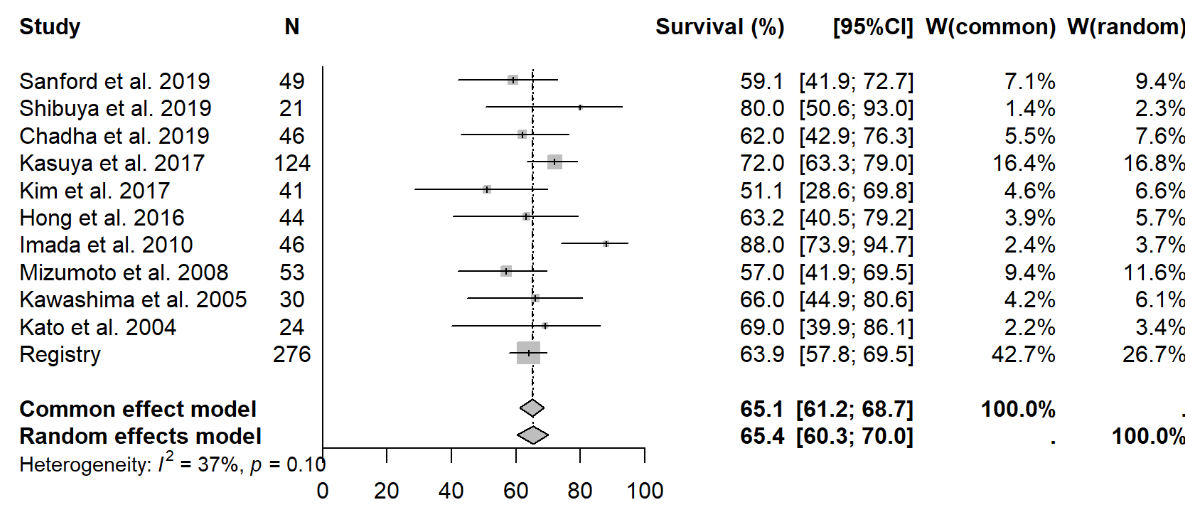


Supplement 11b, 2-year overall survival rate focused on large tumor (SBRT).


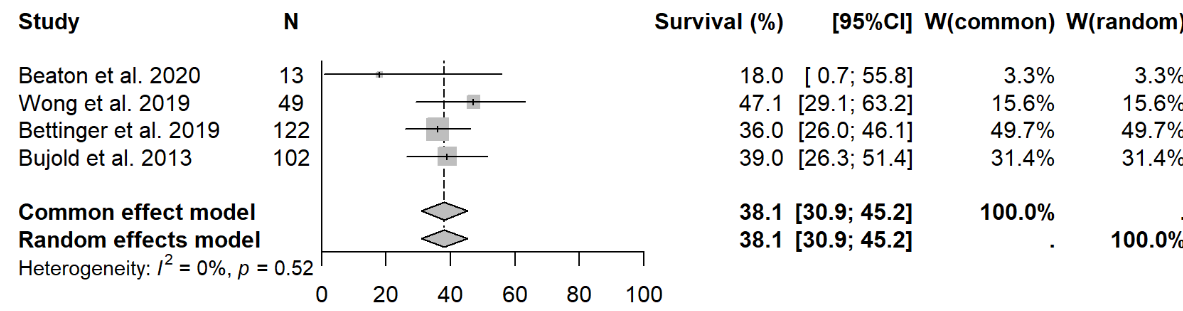


Supplement 11c, 2-year overall survival rate focused on large tumor (3DCRT).


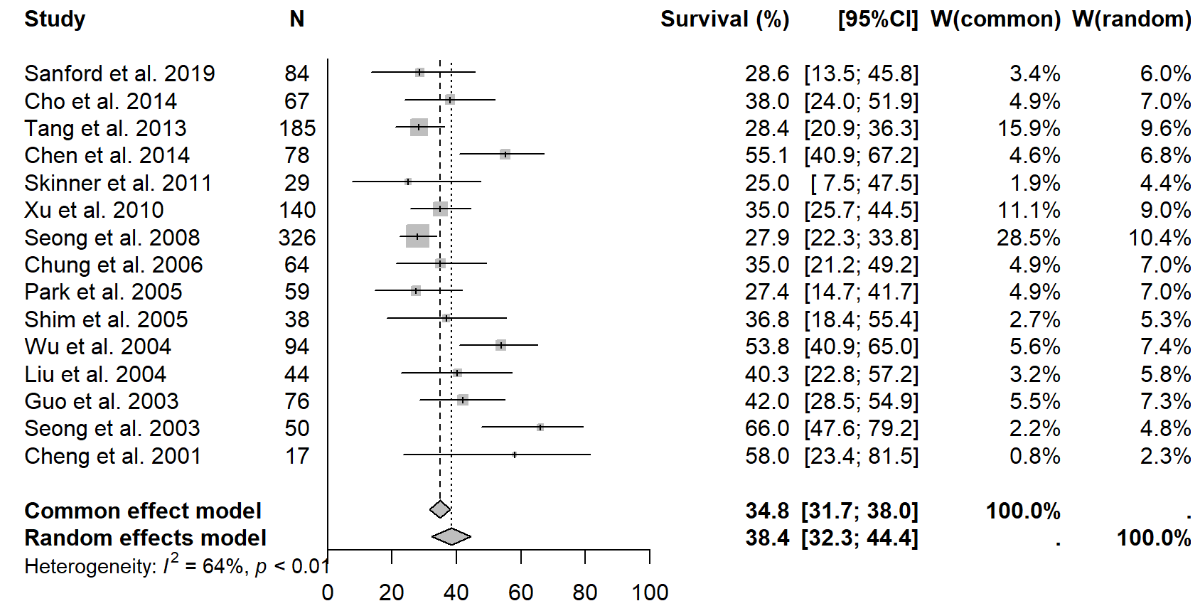

Supplement: Supplementary file 12 — Data S11: Forest plot of 2‐year overall survival rate for each modality (large HCC). [file CAM4-15-e71639-s002.docx]
