# Supplementary material for: Prospective Registry and Meta‐Analysis of Particle Therapy for Hepatocellular Carcinoma: Clinical Outcomes and Real‐World Impact
Source: Cancer Med. 2026 Feb 20;15(3):e71639. doi: 10.1002/cam4.71639 (PMC12921530; doi:10.1002/cam4.71639)

Supplement 12a, 3-year overall survival rate focused on large tumor (Particle Therapy).


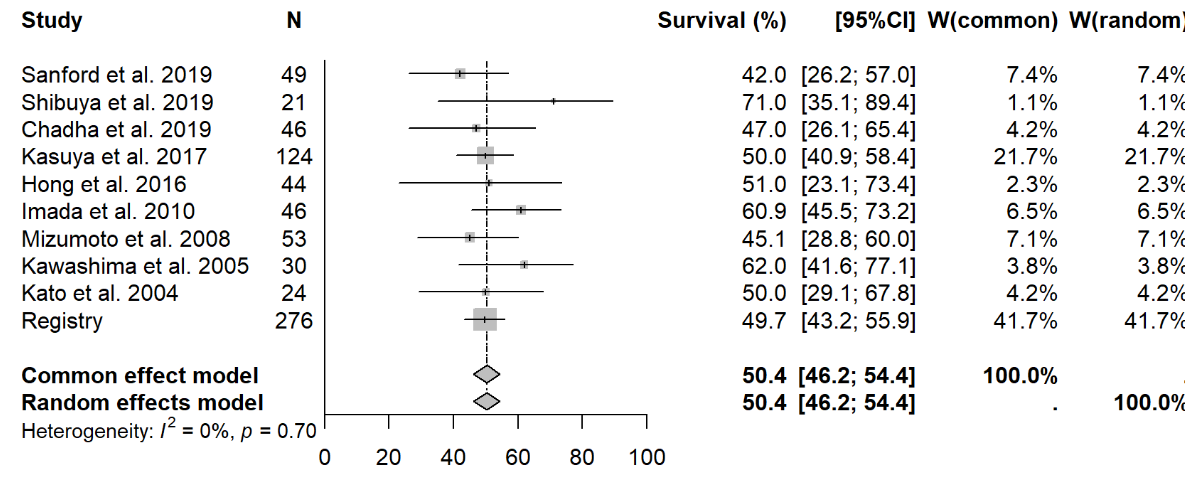


Supplement 12b, 3-year overall survival rate focused on large tumor (SBRT).


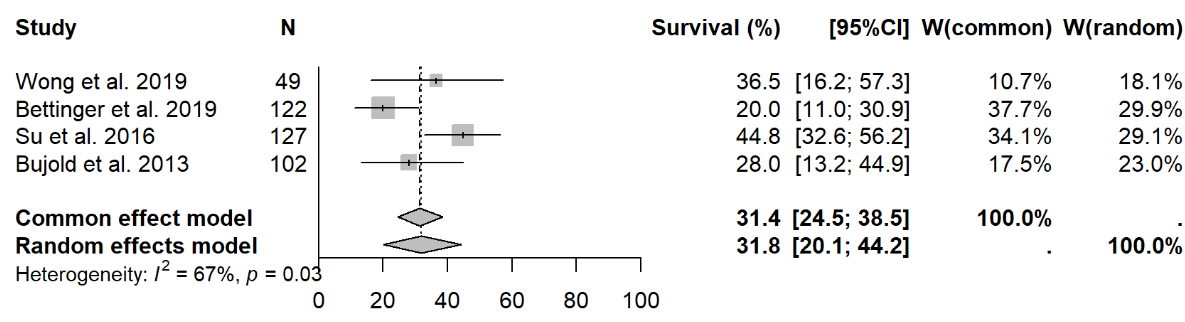


Supplement 12c, 3-year overall survival rate focused on large tumor (3DCRT).


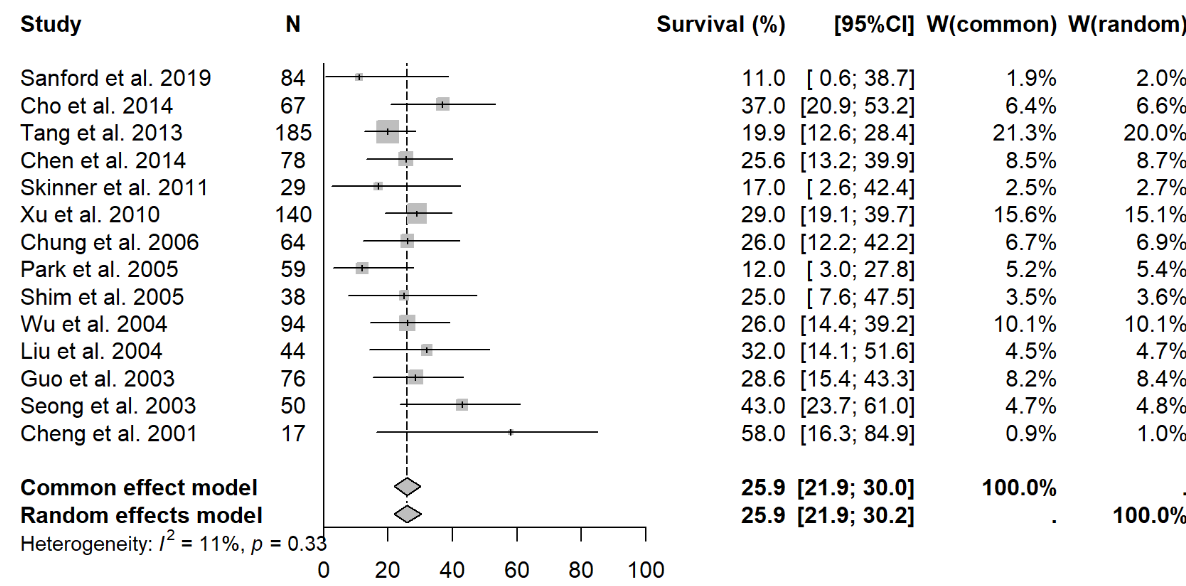

Supplement: Supplementary file 13 — Data S12: Forest plot of 3‐year overall survival rate for each modality (large HCC). [file CAM4-15-e71639-s001.docx]
